# Autism and Hidden Imagination: Raising and Educating Children Who Cannot Express Their Minds

**DOI:** 10.3390/healthcare9020150

**Published:** 2021-02-02

**Authors:** Clair Berube

**Affiliations:** Department of Education, Watts School of Professional Studies, Virginia Wesleyan University, Virginia Beach, VA 23455, USA; cberube@vwu.edu

**Keywords:** autism, imagination, verbal communication, communication disorders, learning styles

## Abstract

This is a reflection on an article written in 2007, entitled Autism and the Artistic Imagination: The Link between Visual Thinking and Intelligence. The author is a parent of a 6-year-old with autism who is now 19 and is non-verbal who has trouble expressing his thoughts, feelings and desires, and discusses some theories behind autism spectrum communication disorders and seeks to understand why there is so much difficulty with communication with some on the spectrum. The 2007 article employed Howard Gardner’s Multiple Intelligence Theory as a framework to discuss the visual and spatial learning abilities of kids with autism, and this update posits that the nonspeaking population of the autism community do indeed have different ways of understanding the world, theories of mind and awareness enough to be able to communicate if only the proper links and opportunities are provided. The lack of communication is not due to a lack of a sense of self, but of a lack of understanding of the neuro-typical community that people with autism are speaking a second language, and need help with the translation.

In 2007, I was the parent of a 6-year-old with autism. I was struggling with a diagnosis that was still pretty fresh, and I was desperately trying to navigate the choppy autism waters. My son John, who is very bright, was (is) a non-verbal boy who has trouble expressing his thoughts, feelings and desires. Being a professor and scholar, of course my natural inclination was to aim that skill set at this autism problem, in the hopes that I cold “crack the code” and help my child connect with the world. That year, I published an article entitled Autism and the Artistic Imagination [1]: The Link Between Visual Thinking and Intelligence, that employed Howard Gardner’s Multiple Intelligence Theory as a framework to discuss the visual and spatial learning abilities of kids with autism, including my son John.

Johnny is the result of a re-marriage in my late 30s; I was 40 when he was born. He is now 19 and in a self-contained SECEP (Southeastern Cooperative Education Programs) class for kids with autism in Virginia where we currently live. He officially graduated from high school last June, but has 4 more years of transition education to prepare him for the world. It has been a long journey since his diagnosis at age 3 in New York at the Weill Cornell Medical Center. I was in New York with my husband and son John, teaching at Wagner College on Staten Island. While I worked, he attended the Wagner College pre-school. It did not go very well and did not last long, with Johnny not making it to Christmas before the staff requested that I withdraw him from the program since he needed more than they could provide. I remember arguing with the staff that he only had something like “Einstein Syndrome”, where he was very smart but would start talking eventually. I was convinced that all would be “normal” as he grew out of his autism. I, of course, was in denial, and confused as to where to place him. He wound up attending a “Special Sprouts” program in Brooklyn, and then a special autism program in one of the public schools. New York City, by the way, has really good special ed services.

During this time frame, I was convinced he would “outgrow” his autism and tried every fad in the book to “cure” him from it. Even though I was writing about accepting him as he was (and I did and currently do), I was also buying $6 loaves of gluten-free bread and limiting his carbs until he was rail thin. This lasted for a couple of years until I decided to experiment with a regular diet again. He did not seem to get worse, and he gained weight. This was a very frustrating time, and wore me down emotionally. When you read books like the ones Jenny McCarthy writes that tell of how she cured her son of autism by sheer will, and the constant talk about the “narrow window” that is available to do this, it makes parents like me want to jump out of that window [2]. Especially for a researcher like myself; it was very humbling not to be able to solve this problem… or at least solve what I thought was a problem. Indeed, not measuring up to another parent who is apparently curing her son, makes one feel like a failure, which is not helpful when you have to have enough strength and confidence to raise a child with autism.

There was a time in New York, where Johnny would say single words from time to time. He would hum tunes that were recognizable. He had good eye contact, was funny, and had a sense of irony. So, I could be forgiven for thinking that he would grow out of it. He would gesture to me if he wanted something, still not speaking consistently. He also stopped talking altogether eventually. When I say that Johnny is “non-verbal”, I do not mean that he is not noisy. He verbalizes all of the time; he just does not speak English or any language for that matter. He has verbalizations when he is happy, upset, sad, etc. However, he will not speak in words or sentences.

During this time, we also moved to Chicago for a semester for a visiting professor position, but was dumbstruck at the horrible lack of special education options there. Johnny went from a full day of school in New York, to two mornings a week in Chicago. We did not last there long and went back to Virginia. Lots of academics move for jobs, and it was a peek into the dizzying disparities of resources between states and districts. It is not unheard of for parents of kids with autism to uproot and move across country for schools that have decent services. The injustice of special education includes the fact that neuro-typical kids have an array of choices of schools they can attend, including public and private. Special education kids on the other hand, only have their local public school in the majority of cases. Even if a family is lucky enough to live near a private school for autism, the cost can be exorbitant. There is an injustice in that. Summer camps have this same disparity; a neuro-typical kid can have his or her pick of summer camps, but kids with autism have almost no options. If there are camps available, like there was for my son for a few years, the funds can dry up the camps disappear, and there is not a plan B. Johnny’s favorite summer camp, Camp Horizon, lost funding with budget cuts this summer after 20 years of helping kids and young adults enjoy themselves at camps like other kids do. The United Way funding that had supported the camp was eliminated, and kids like Johnny had nothing to do last summer. The camp was cancelled in February. Autism camps are few and far between with limited choices to help kids like John have normal childhood experiences. Driving home one day after picking John up from camp, he took my hand in the car and kissed the back of it. He had never done anything like that before.

## 1. Official Diagnosis

Since the return to Norfolk, Virginia in 2005, Johnny has been officially diagnosed as having Autism Spectrum Disorder. As recently as 2019 he underwent psychometric evaluation in order to have documentation on file for my quest to obtain legal guardianship of him, which was finalized in 2020. The psychology practice where he was evaluated conducted interviews with me, observations of my son while in the same room physically, and conducted the following screenings: 

ADOS-2: Autism Diagnostic Observation Schedule, Second Edition [3]

ASRS: Autism Spectrum Rating Scale

CBCL: Child Behavior Checklist

CTONI-2: Comprehensive Test of Nonverbal Intelligence, Second Edition [4]

PPVT-4: Peabody Picture Vocabulary Test, Fourth Edition

TRF: Teacher Report Form

Vineland-3: Vineland Adaptive Behavior Scales, Third Edition, Parent/Caregiver Form [5]

Johnny was found to have profound intellectual disability, which was not surprising, given his history. The severity of his condition made it impossible to accurately measure IQ, however his deficits include the areas of affective, somatic and thought problems. His adaptive functioning and communication, daily living skills and socialization based on the ABC instrument are all in the range of 20 with 100 being the norm. This score is different than an IQ score in that it measures several domains, including those mentioned previously, however it is still much lower than the mean. He was scored on the Autism testing instruments (ADOS-2 & ASRS) as having Autism Spectrum Disorder, level 3, which is considered severe autism that interferes with the ability to provide self-care and requires around-the-clock care. Even with these diagnoses, Johnny has many strengths and his teachers since elementary school say he is very smart. This is the mystery of autism, and trying to quantify it, especially since it is a spectrum disorder, can be very frustrating. 

## 2. Communication

Over the years, Johnny has used several methods to communicate, and among the first formal systems was PECS (picture exchange communication system). PECS [6] are a really easy for kids to be able to express what they want and need. There is a binder with Velcro and several little pictures with Velcro as well. A child will remove the picture and hand it to the adult who can help them get the thing they want.

**Figure 1:**
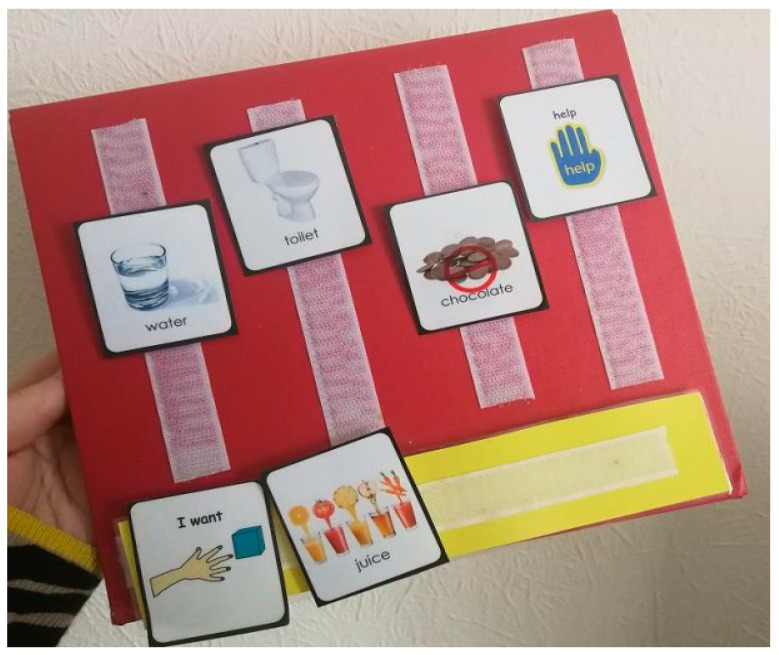
Communicating Differently V&A Blog (vam.ac.uk) [8].

“The PECS teaching protocol is based on B.F. Skinner’s book [7], Verbal Behavior, and broad spectrum applied behavior analysis (Figure 1) Specific prompting and reinforcement strategies that will lead to independent communication are used throughout the protocol. The protocol also includes systematic error correction procedures to promote learning if an error occurs. Verbal prompts are not used, thus building immediate initiation and avoiding prompt dependency. PECS consists of six phases and begins by teaching an individual to give a single picture of a desired item or action to a “communicative partner” who immediately honors the exchange as a request. The system goes on to teach discrimination of pictures and how to put them together in sentences. In the more advanced phases, individuals are taught to use modifiers, answer questions and comment. The primary goal of PECS is to teach functional communication. Research has shown that some learners using PECS also develop speech. Others may transition to a speech generating device (SGD). The body of research supporting the effectiveness of PECS as an evidence-based practice is substantial and continues to expand, with more than 150 research articles from all over the world” [7].

**Figure 2:**
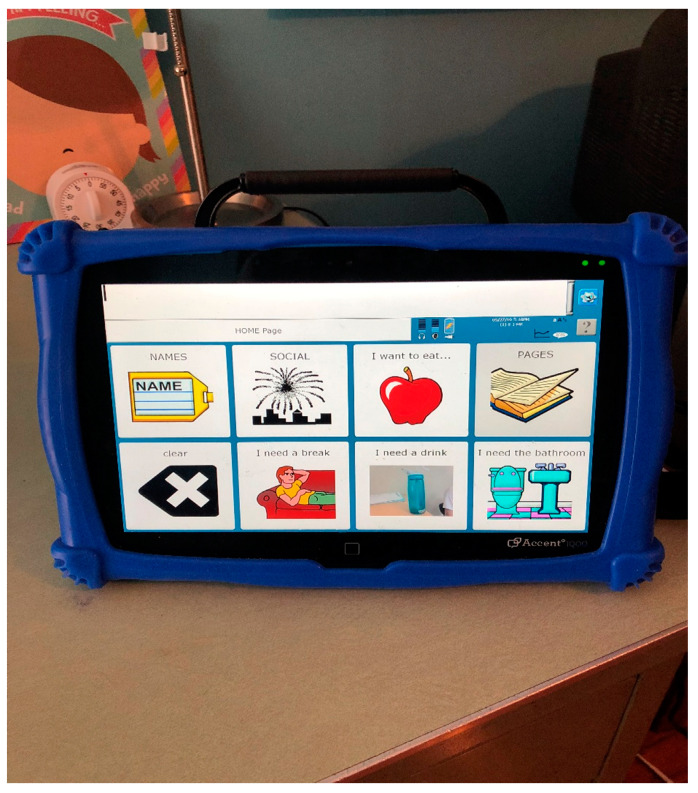
Theories of Language Dysfunction in Autism.

Johnny still uses PECS on a regular basis, and he also uses an “aug-com” device (Augmentative Communication). These devices are like large iPads and they talk for the child. Johnny uses an Accent 1000 device, which has pictures that talk when pressed (Figure 2).

Communication obviously infers that the passing of information is occurring between two people. However, if one of the communication partners is not verbally communicating, or is using his or her “own language” to communicate, how can this transfer or passing of information take place? Many theories have been developed over the years as we have grown to understand autism. Some theories say that people with autism have no empathy, which is needed for communication. Fletcher-Watson and Bird [9] argue that this notion is false, and the problem might be due to the fact that autistics have a hard time understanding neruo-typicals, due to the fact that they show higher empathy with other autistics. Empathy may not the issue, but understanding may be Fletcher-Watson and Bird [8], quote a study by Crompton, Fletcher-Watson, and Ropar [9] that stated “Very recent research shows that when we examine the interactions of two autistic people, we see higher rapport than for autistic/non-autistic pairs, both as rated by people in the interaction, and by naïve observers” [10]. Other theories have stated that people with autism have no personal theory of “mind”… or “lack an internal language”, which is also necessary for successful communication (source). According to Pedreno, et al. [11], “Theory of Mind (ToM) refers to the ability to ascribe mental states to oneself and others in order to understand and predict their behaviour. It has its origins in the pioneering work of Premack and Woodruff [12] in primatology, and it has been found to be a crucial ability for social adaptation. This ability has also been referred to as “mentalizing [13] or “mind reading” [14]. In the field of psychopathology, the study of ToM was initially focused on Autism Spectrum Disorders (ASD), as a way to explain the social and communication problems defining these disorders. There is now consistent evidence that subjects with ASD show a clear difficulty in their ability to understand the nature of mental representations and the role that these play in determining people’s behaviour [14,15,16,17,18,19] Perhaps it is arrogant for neuro-typical people to just assume that people with autism do not have a theory of mind, or are not self-reflective. It may also be arrogant to assume that just because someone cannot score well on a typical verbally based intelligence test, they are not intelligent.

However, what if people with autism have their own language? Like a first language…meaning that learning the language of the culture they live in would be almost like learning a second language? Bogdashina [20] posited that people with autism speak a different language than the rest of us. According to Bogdashina: “We may hypothesize that autistic children (or at least some of them) ‘speak’ (even those who are mute) a different language. Verbal language is a sort of foreign language for them. As they do not learn it naturally earlier in their lives, we have to help them master their second language with the support of their ‘first language’ if we want to share a means of communication with them. So, what language do autistic people speak? Furthermore, can we talk about any language at all in the case of non-verbal people? The answer is affirmative. Non-verbal people do possess their own language system, external and internal speech. Before we can teach them a ‘foreign language’ we have to learn theirs first in order to get the ability to ‘interpret/translate’ their messages at the initial stages of our communication with them” (p. 32). This is a very interesting concept, and makes sense in many ways. 

Bogdashina [20] argues with the lack of theory of mind hypothesis, and cites O’Neill [21] and Williams [22] as saying that: “Autistic individuals emphasize that all autistic people have a form of inner language even if they cannot communicate through conventional systems such as typing, writing or signing” [20]. It has been posited that autistic individuals think in “pictures” rather than words, and that may be their first language. Temple Grandin is perhaps the world’s most famous person with autism. She is the subject of HBO movies and is the author of several books, and is a professor of animal science at Colorado State University. Dr. Grandin has become a successful academic in spite of her autism (in this cause *because* of her autism) and has designed a system of leading cattle to slaughter that is a more humane system than had been previously used…and she got the idea of “squeezing” cattle through a series of chutes leading them through a path to the slaughter house because squeezing had always brought her comfort as a child with autism. She discovered that when cattle feel pressure on their bodies as they walk to slaughter, their stress levels lower, creating less stress chemicals in the resulting meat, and more importantly to her, less stress in the cattle. Grandin herself has spoken of how she sees the world:

“I think in pictures. Words are like a second language to me. I translate both spoken and written words into full-color movies, complete with sound, which run like a VCR tape in my head. When somebody speaks to me, his words are instantly translated into pictures. Language-based thinkers often find this phenomenon difficult to understand, but in my job as an equipment designer for the livestock industry, visual thinking is a tremendous advantage.

Visual thinking has enabled me to build entire systems in my imagination. During my career I have designed all kinds of equipment, ranging from corrals for handling cattle on ranches to systems for handling cattle and hogs during veterinary procedures and slaughter. I have worked for many major livestock companies. In fact, one third of the cattle and hogs in the United States are handled in equipment I have designed. Some of the people I’ve worked for don’t even know that their systems were designed by someone with autism. I value my ability to think visually, and I would never want to lose it. One of the most profound mysteries of autism has been the remarkable ability of most autistic people to excel at visual spatial skills while performing so poorly at verbal skills. When I was a child and a teenager, I thought everybody thought in pictures. I had no idea that my thought processes were different. In fact, I did not realize the full extent of the differences until very recently. At meetings and at work I started asking other people detailed questions about how they accessed information from their memories. From their answers I learned that my visualization skills far exceeded those of most other people.

I credit my visualization abilities with helping me understand the animals I work with. Early in my career I used a camera to help give me the animals’ perspective as they walked through a chute for their veterinary treatment. I would kneel down and take pictures through the chute from the cow’s eye level. Using the photos, I was able to figure out which things scared the cattle, such as shadows and bright spots of sunlight. Back then I used black-and-white film, because twenty years ago scientists believed that cattle lacked color vision. Today, research has shown that cattle can see colors, but the photos provided the unique advantage of seeing the world through a cow’s viewpoint. They helped me figure out why the animals refused to go in one chute but willingly walked through another” [23].

This excerpt is from Dr. Grandin’s website and is taken from maybe her most famous book, Thinking in Pictures; My Life with Autism [24]. This was a ground-breaking look into the mind of an autistic adult. The internal languages autistic people understand are different than those of the neuro-typical world. It is no wonder that there is a problem with communication, as they go through school, try to secure jobs, and try to be understood in the larger world. In Nagle’s [25] forward to the book Innovative Investigations of Language in Autism Spectrum Disorder, she states that one quarter of all children with autism are non-verbal, and that modern research shows that those who are verbal (and are fluent) do not show markedly different defects in speech than the normal population (xii). Why these many children with autism still cannot speak even after extensive therapy is still a mystery. However, it is becoming clear that they do have their own way of understanding the world. ABA therapy has been shown to help many children with autism towards the goal of communicating. ABA stands for Applied Behavioral Analysis. According to the Association for Science in Autism Treatment, “ABA is effective in increasing behaviors and teaching new skills (National Autism Center [NAC]”, [26,27] In addition, many studies demonstrate that ABA is effective in reducing problem behavior [26]. A number of studies also indicate that, when implemented intensively (more than 20 h per week) and early in life (beginning prior to the age of 4 years), ABA may produce large gains in development and reductions in the need for special services [28]; however, large studies with strong experimental designs are needed to confirm the results reported for intensive, early intervention. The United States Surgeon General [29] concluded, “Thirty years of research demonstrated the efficacy of applied behavioral methods in reducing inappropriate behavior and in increasing communication, learning and appropriate social behavior” [30]. My son John had in-home ABA therapy for two years and we saw gains in his ability to communicate with his augmentative communication device (a large tablet that has a program that speaks for him) and in his ability to deal with frustration, among other things.

## 3. Communication Reimagined

During a typical day of John Berube, my almost 19-year-old young man with autism, his voice is going all the time. I can tell which sounds are happy, which are distressed, and which convey contentment. However, there is no language; none at all. I have noticed lately that he plays around with sounds more, like saying mamamama or babababa at times. Sometimes when he does this it is quiet, like he is practicing and does not want anyone to hear. I cannot say for sure what is going on in that mind of his, but he is a very noisy child and I can pick out his “voice” from a roomful of people. I can hear him whine from another room as if to say he wants something and does not know how to ask for it. He does not initiate bathroom breaks, but will go if taken and if he needs to go. Thy mystery of why it is so difficult to toilet train people with autism is still being studied, and might have to do with a lack of connection between brain and body. Kroeger and Sorensen [31] state that it may also be due to both a lack of communication skills and the inability to generalize specific learning examples to more generalized situations and that these considerations need to be taken into account during the implementation of training programs (2010). John does not really like using his aug-com device, but does communicate with me on it if he is in a good enough mood. He uses PECS more often than the device, because it is easier, and what teenager does not want to go the easier route? He does many things that I tend to blame on autism, but could be solely attributed to being a cranky teenager. There is a VENN diagram of behaviors with a middle section uniting the two and sometimes it is very hard to distinguish. I am sure he is somewhat spoiled, because when he whines or cries for some reason, is it because he is being difficult, or is he in medical distress?

On top of everything else, on 26 July 2019 he had a grand mal seizure for the first time and had to go on the anti-convulsant drug Keppra. This drug is the one his neurologist’s daughter would be on if she had seizures I was told, and has almost no side effects except for a common one of irritability. John was already experiencing teenage irritability so we were discouraged to hear this. He started the drug and had a bad month of adjustment, including hitting himself so hard on the chin that he chipped a bottom tooth. He hits himself on the chin and bites his hands if he is really upset, and he has had scars on his hands and bruising on his chin off and on for a couple of years now. The side-effects have calmed down and he seems back to his happy self most of the time, but there are still times when he awakes at 3 am crying for reasons I do not know. That is when I try to comfort him, rub his arm or his hair and sing him back to sleep. I ask him what is wrong, and sometimes in the wee hours I turn on his aug-com device but it is not sufficient communication for the moment. The next morning, if he feels better, he will look into my eyes and smile and he seems to be saying “thank you” for putting up with me last night. Sometimes he can be so cranky and carrying on so much that he starts giggling at his own theatrics, as if even he knows how ridiculous he is acting. Theory of mind? This is evidence of self-awareness. There does appear to be a connection between his lack of ability to communicate and his self-injurious behaviors. As he has grown and become a young adult, it has become worse. How much self-injury is directly related to his lack of ability to communicate is conjecture on our part, but definitely an IEP transition goal to be addressed in his remaining years of school.

## 4. What Can Parents/Caregivers Do?

There are things we as caretakers can do to ensure our loved ones with autism are communicating as well as they possibly can be. Dawson and Elder [32] provide some useful advice:

1. Encourage play and social interaction. Children learn through play, and that includes learning language. Interactive play provides enjoyable opportunities for you and your child to communicate. Try a variety of games to find those your child enjoys. Additionally, try playful activities that promote social interaction. Examples include singing, reciting nursery rhymes and gentle roughhousing. During your interactions, position yourself in front of your child and close to eye level—so it is easier for your child to see and hear you.
2. Imitate your child. Mimicking your child’s sounds and play behaviors will encourage more vocalizing and interaction. It also encourages your child to copy you and take turns. Make sure you imitate how your child is playing—so long as it is a positive behavior. For example, when your child rolls a car, you roll a car. If he or she crashes the car, you crash yours too. However, do not imitate throwing the car!
3. Focus on nonverbal communication. Gestures and eye contact can build a foundation for language. Encourage your child by modeling and responding these behaviors. Exaggerate your gestures. Use both your body and your voice when communicating—for example, by extending your hand to point when you say “look” and nodding your head when you say “yes”. Use gestures that are easy for your child to imitate. Examples include clapping, opening hands, reaching out arms, etc. Respond to your child’s gestures: When she looks at or points to a toy, hand it to her or take the cue for you to play with it. Similarly, point to a toy you want before picking it up.
4. Leave “space” for your child to talk. It is natural to feel the urge to fill in language when a child does not immediately respond. However, it is so important to give your child lots of opportunities to communicate, even if he is not talking. When you ask a question or see that your child wants something, pause for several seconds while looking at him expectantly. Watch for any sound or body movement and respond promptly. The promptness of your response helps your child feel the power of communication.
5. Simplify your language. Doing so helps your child follow what you are saying. It also makes it easier for her to imitate your speech. If your child is nonverbal, try speaking mostly in single words. (If she’s playing with a ball, you say “ball” or “roll”.) If your child is speaking single words, up the ante. Speak in short phrases, such as “roll ball” or “throw ball”. Keep following this “one-up” rule: Generally use phrases with one more word than your child is using.
6. Follow your child’s interests. Rather than interrupting your child’s focus, follow along with words. Using the one-up rule, narrate what your child is doing. If he’s playing with a shape sorter, you might say the word “in” when he puts a shape in its slot. You might say “shape” when he holds up the shape and “dump shapes” when he dumps them out to start over. By talking about what engages your child, you’ll help him learn the associated vocabulary.
7. Consider assistive devices and visual supports. Assistive technologies and visual supports can do more than take the place of speech. They can foster its development. Examples include devices and apps with pictures that your child touches to produce words. On a simpler level, visual supports can include pictures and groups of pictures that your child can use to indicate requests and thoughts. For more guidance on using visual supports, see Autism Speaks ATN/AIR-P Visual Supports Tool Kit [32].
8. I have done all of these suggestions, including imitating him when he is verbalizing. The imitation of his verbalizations is sometimes met with a smile from him, as if he is appreciative of my efforts on his behalf, however my understanding of how he feels are my own. I cannot really know what he is thinking most of the time.

Your child’s therapists are uniquely qualified to help you select and use these and other strategies for encouraging language development. Tell the therapist about your successes as well as any difficulties you are having. By working with your child’s intervention team, you can help provide the support your child needs to find his or her unique “voice” [32].

## 5. Conclusions

My hope is that one day Johnny will have a pretty secure way of communicating with his caretakers. His Medicaid waiver allows him to move out in a few years to his own place or one shared with others like himself, with 24-h care. He can come stay with me for visits whenever I see fit, but it will be nice for him to be out in the world being his best version of the man he will be. I hope to have him resume some home-based ABA therapy soon as that helps a lot with his practical skills. He recently had a couple of years of ABA therapy and it really helped him be a better communicator. In the end though, the spectrum of autism can be dizzying, as there are no two people with autism who are alike. I have learned to “translate” Johnny’s wants and desire to various aides who come to our home to help with him… but he needs to bridge that gap so he can convey his needs to them himself. He has come a long way but has a long way to go, as do we all. Helping these kids connect with the world is a noble endeavor and one I hope researchers keep at so they can crack the code. There is “someone” in that head of his, and the personality that shines through reminds me that we cannot be so arrogant to suggest that because someone cannot communicate with you the way you are use to, then that means that there is either no one in there, or that the person in there is sub-human or lacking human emotions. We cannot continue to de-humanize people with autism by taking away their dignity in this way. One day, the link between how they see the world and the way we see the world will be discovered. Until then, accepting him as a loved son is all I can do.

## Data Availability

Not applicable.

## References

[1] Berube C.T. Autism and the Artistic Imagination: The Link between Visual Thinking and Intelligence. (2007) Article 1. http://escholarship.bc.edu/education/tecplus/vol3/iss5/art1.

[2] McCarthy J. (2007). Louder than Words.

[3] Lord C., Rutter M. (2012). ADOS-2: Autism Diagnostic Observation Schedule.

[4] Hammill D., Pearson N., Weiderholt J. (2009). Comprehensive Test of Nonverbal Intelligence.

[5] Skinner B.F. (1957). Verbal Behavior.

[6] Vineland Adaptive Behavior Scales, Third Edition (Vineland-3). https://images.pearsonclinical.com/images/Assets/vineland-3/Vineland-3-Flyer.pdf.

[7] Picture Exchange Communication System; What is PECs?. https://pecsusa.com/pecs/.

[8] (2009). Communicating Differently. Victoria and Albert Museum V&A Blog. https://www.vam.ac.uk/blog/museum-life/va-faces.

[9] Fletcher-Watson S., Byrd G. (2019). Autism and empathy: What are the real links?. Autism.

[10] Crompton C.J., Ropar D., Evans-Williams C.V., Flynn E.G., Fletcher-Watson S. (2020). Autistic peer to peer information transfer is highly effective. Autism.

[11] Pedreno C., Pousa E., Navarro J.B., Pamias M., Obiols J.E. (2017). Exploring the components of advanced theory of mind in autism spectrum disorder. J. Autism Dev. Disord..

[12] Premack D., Woodruff G. (1978). Does the chimpanzee have a “theory of mind’’?. Behav. Brain Sci..

[13] Frith U., Morton J., Leslie A.M. (1991). The cognitive basis of a biological disorder autism. Trends Neurosci..

[14] Baron-Cohen S. (1995). Mind Blindness: An Essay on Autism and Theory of Mind.

[15] Baron-Cohen S., Leslie A.M., Frith U. (1985). Does the autistic child have a “theory of mind”?. Cognition.

[16] Happé F. (1994). An advanced test of theory of mind: Understanding of story characters thoughts and feelings by able autistic, mentally handicapped and normal children and adults. J. Autism Dev. Disord..

[17] Heavey L., Phillips W., Baron-Cohen S., Rutter M. (2000). The awkward moments test: A naturalistic measure of social understanding in autism. J. Autism Dev. Disord..

[18] Klin A. (2000). Attributing social meaning to ambiguous visual stimuli in higher—Functioning autism and Asperger syndrome: The social attribution task. J. Child Psychol. Psychiatry.

[19] Wellman H.M., Cross D., Watson J. (2001). Meta-Analysis of theory-of-mind development: The truth about false belief. Child Dev..

[20] Bogdashina O. (2005). Communication Issues in Autism and Asperger Syndrome: Do We Speak the Same Language.

[21] O’Neill J.L. (1999). Through the Eyes of Aliens: A Book about Autistic People.

[22] Williams D. (1996). Autism: An Inside-Out Approach.

[23] Grandin T. (2006). Thinking in Pictures: My Life with Autism.

[24] Autism Spectrum Rating Scales. https://www.pearsonassessments.com/store/usassessments/en/Store/Professional-Assessments/Behavior/Autism-Spectrum-Rating-Scales/p/100000354.html#:~:text=The%20Autism%20Spectrum%20Rating%20Scales,ASDs)%20in%20children%20and%20adolescents.

[25] Nagle L. (2016). Innovative Investigations of Language in Autism Spectrum Disorder.

[26] Randolph M.A. (2015). Findings and Conclusions: National Standards Project, Phase 2.

[27] Wong C., Odom S.L., Hume K.A., Cox A.W., Fettig A., Kurcharczyk S., Brock M.E., Plavnick J.B., Fleury V.P., Schultz T.R. (2015). Evidence-Based practices for children, youth, and young adults with autism spectrum disorder: A comprehensive review. J. Autism Dev. Disord..

[28] Reichow B. (2012). Overview of meta-analyses on early intensive behavioral intervention for young children with autism spectrum disorders. J. Autism Dev. Disord..

[29] United States Surgeon General (1998). Mental Health: A Report of the Surgeon General.

[30] (2020). Applied Behavior Analysis (ABA)—Association for Science in Autism Treatment. asatonline.org.

[31] Kroeger K., Sorensen R. (2010). A parent training model for toilet training children with autism. J. Intellect. Disabil. Res..

[32] Dawson G., Elder L. (2013). Helping Your Child with Nonspeaking Autism Talk|Autism Speaks. https://www.autismspeaks.org/expert-opinion/seven-ways-help-your-child-nonverbal-autism-speak.

